# Clinical features of progressive encephalomyelitis with rigidity and myoclonus: Case Report

**DOI:** 10.3389/fimmu.2025.1616207

**Published:** 2025-10-30

**Authors:** Lixia Qin, Qihua Chen

**Affiliations:** ^1^ Department of Neurology, The Second Xiangya Hospital, Central South University, Changsha, Hunan, China; ^2^ Clinical Medical Research Center for Stroke Prevention and Treatment of Hunan Province, Department of Neurology, The Second Xiangya Hospital, Central South University, Changsha, China

**Keywords:** progressive encephalomyelitis with rigidity and myoclonus, stiff person syndrome, glycine receptor, GAD65, brainstem dysfunction

## Abstract

Progressive encephalomyelitis with rigidity and myoclonus (PERM) is a rare neurological disorder characterized by rigidity, painful spasms, hyperekplexia, brainstem involvement, and autonomic dysfunction. In China, limited awareness and delayed antibody testing often hinder early diagnosis. Here, we report four cases of PERM, three of which required intensive care unit (ICU) admission. The clinical and immunological features of these patients were systematically summarized. This case series highlights the characteristic clinical and immunological profiles of PERM and underscores the importance of early recognition and timely intervention, as most patients achieve substantial recovery with immunosuppressive therapy.

## 1 Introduction

Stiff-person syndrome (SPS) is characterized by episodes of fluctuating muscle rigidity and painful spasms, which can occur spontaneously or be triggered by various stimuli (1, 2). The immune mechanisms underlying SPS involve autoantibodies targeting proteins predominantly expressed at inhibitory synapses. To date, six key autoantigens have been identified: glutamic acid decarboxylase (GAD65), the α1-subunit of the glycine receptor (GlyR), amphiphysin, gephyrin, dipeptidyl peptidase-like protein 6 (DPPX), and the γ-aminobutyric acid-A receptor (GABAAR).

In 1976, Whiteley et al. described two cases of encephalomyelitis presenting with muscular rigidity and stimulus-sensitive spasms, distinct from classic SPS (3). Progressive encephalomyelitis with rigidity and myoclonus (PERM) is characterized by hyperekplexia, myoclonus, and dysfunction involving the brainstem, pyramidal tracts, sensory pathways, and autonomic nervous system (4). PERM represents a more severe variant of SPS (5). In an early series of PERM cases, GAD antibodies were detected (6). Since the first description in 2008 of a patient with PERM who tested positive for GlyR antibodies (7), additional cases with GlyR antibodies have been reported. The pathogenic role of GlyR antibodies is further supported by passive antibody transfer models (5). It is generally thought that PERM is mainly associated with GlyR antibodies, whereas classic SPS or stiff-limb syndrome (SLS) with or without overlapping syndromes more frequently occurred with GAD65 antibodies (4, 8). There is currently no consensus as to whether PERM should be classified within SPS spectrum disorders or regarded as a distinct clinical entity (9, 10).

This study retrospectively reviewed the clinical data of four PERM patients treated at our center, with the objective of analyzing and summarizing their clinical and immunological features. The findings aim to enhance clinicians’ awareness of PERM, improve diagnostic accuracy and treatment protocols, and lay the groundwork for standardized management strategies for this condition.

## 2 Materials and methods

### 2.1 Standard protocol approvals, registrations, and patient consents

Informed consent was obtained from all participants following the study’s approval by the Ethics Committee of the Second Xiangya Hospital, Central South University, Changsha, China.

### 2.2 Clinical data

Clinical records and photographs were reviewed. Patients were recontacted for clinical re-evaluation and antibody detection.

### 2.3 GlyR and GAD65 antibodies detection

The GlyR and GAD65 antibodies were detected using a cell-based assay (CBA). To identify target antibodies in patient samples, human target genes were cloned into pcDNA3.1 vectors and transfected into HEK293 cells. Following a 24-hour transfection period, the cells were fixed with 4% paraformaldehyde for five minutes and washed with phosphate-buffered saline (PBS) containing 0.1% Tween 20. The fixed cells were then prepared for antibody detection. For the assay, patient serum (diluted 1:10 in 0.4% PBS-Triton X-100) and undiluted CSF were incubated with the transfected cells for one hour at room temperature. After incubation, the cells were washed three times with PBS-0.1% Tween 20 and subsequently incubated with FITC-conjugated goat anti-human immunoglobulin G (IgG) for 30 minutes at room temperature. The cells were washed again in PBS-0.1% Tween 20 before analyzing reactivity using immunofluorescence microscopy. HEK293 cells transfected with empty vector plasmids were used as controls, and all samples were independently evaluated by two investigators. Antibody titers were determined through serial dilutions of the serum and CSF (ranging from 1:10 to 1:1000) for samples that tested positive. The titer was defined as the highest dilution at which specific fluorescence of HEK293 cells remained visible.

## 3 Results

### 3.1 Patients characteristics

**Figure 1:**
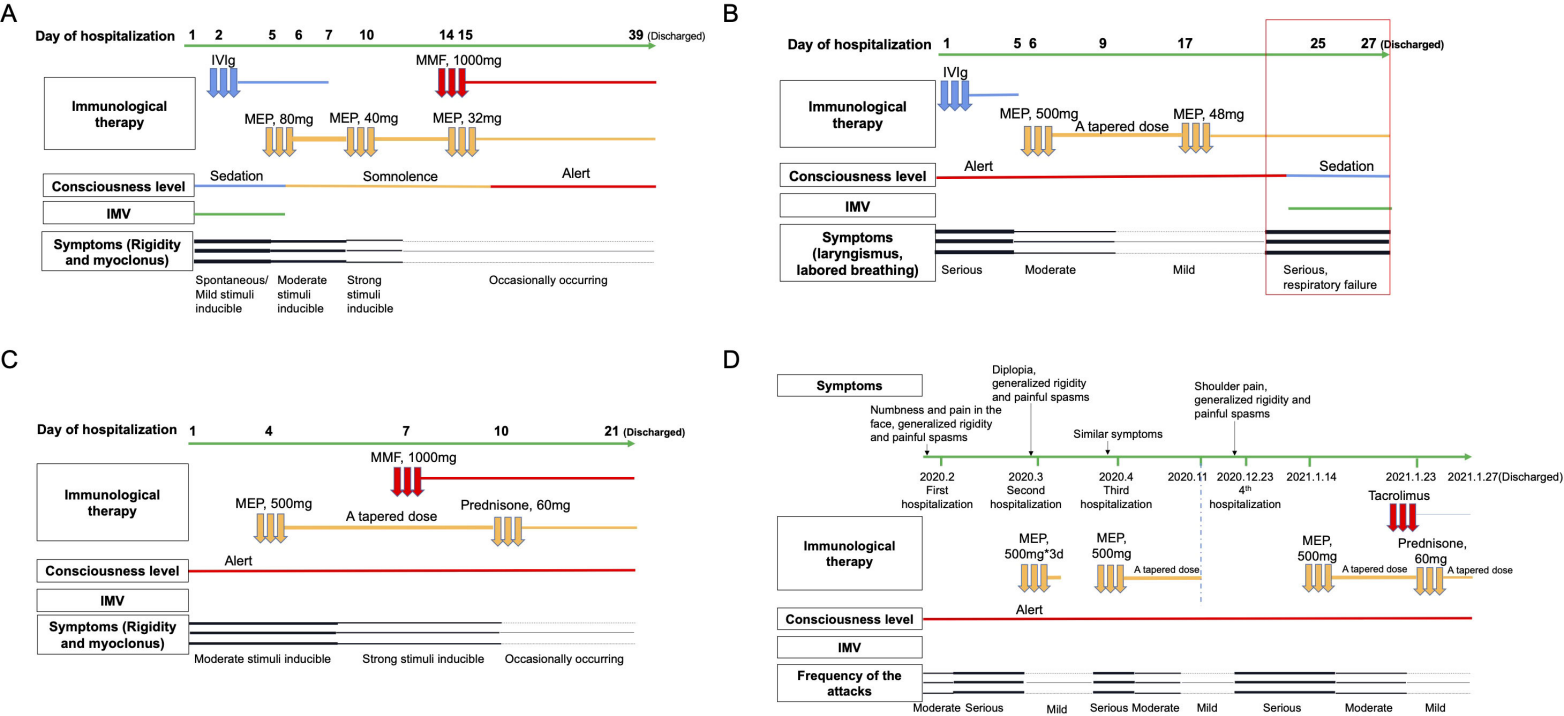
Clinical course of the four patients with PERM. Symptoms, IMV, consciousness level, and immunological therapy administered during the patients’ hospitalization are displayed. Panel **(A)** shows clinical course of Case 1, panel **(B)** Case 2, panel **(C)** Case 3, and panel **(D)** Case 4. IMV, invasive mechanical ventilation. IVIg, intravenous immunoglobulin. MEP, methylprednisolone. MMF, mycophenolate mofetil.

Four patients with PERM were identified, including three males and one female. The mean age at onset was 58.75 ± 9.26 years (range: 44–68), with males comprising 75% of the cases. Among the four patients, no significant medical history was reported, except for one case with a history of myasthenia gravis (MG) associated with thymoma. The most characteristic symptoms observed in all cases were unexplained stiffness and spasms. Diagnosis in all cases was confirmed by the presence of positive antibodies. The clinical features of the four patients are summarized in **Table 1**. Clinical course of the four patients with PERM is shown in **Figure 1**.

**Table 1:** Clinical features of the four patients with PERM.

Clinical characteristics	Case 1	Case 2	Case 3	Case 4
Gender, age (years) at onset	M, 65	M, 68	M, 58	F, 44
Onset of Disease	Acute onset	Acute onset	Acute onset	Subacute onset
Time to diagnose	13 days	11 days	9 days	11 months
Antibodies (titer)	Serum GlyR (1: 10); CSF GlyR (1: 10)	Serum GlyR (1:30); CSF GlyR (1:10); Serum GAD (1:10)	Serum GlyR (1: 10); CSF GlyR (1: 10)	Serum GlyR (1:10); CSF GlyR (1:10); Serum GAD65 (1:30); CSF GAD65 (1:30)
Combined antibodies	Serum SSA, Ro-52	Serum MOG antibody	–	Serum AchR and Titin
Tumor	–	–	–	Thymoma
Symptoms	Stiffness, spasms, respiratory failure	Laryngismus (trismus, dysphagia, alalia, dribbling, bucking, respiratory failure)	Rigidity and spasms in both lower limbs	Numbness in right face, diplopia, shoulder pain, startle-induced episodes of generalized rigidity and painful spasms in the face, trunk and limbs
Brain/spinal cord MRI	–	–	–	–
CSF routine examination	Slightly increased white cell count	Slightly increased protein, increased glucose, and decreased chloride	Slightly elevated glucose levels	–
EEG	Diffuse slow-wave abnormalities	Diffuse slow-wave abnormalities	Diffuse slow-wave abnormalities	Not performed
EMG	MUP	Not performed	Axonal degeneration, demyelination, Neurogenic abnormalities, early denervation changes, MUP	–
Treatment	IVIg, MEP followed by tapering oral prednisone, MMF	IVIg, MEP	MEP followed by tapering oral prednisone, MMF	IVIg MEP followed by tapering oral prednisone, tacrolimus
Outcome	Substantial improved	Short-term improvement followed by deterioration	Substantial improved	Complete recovery

M, male. F, female. CSF, cerebrospinal fluid. MRI, magnetic resonance imaging. EEG, electroencephalogram. EMG, electromyography. MUP, motor unit potential. IVIg, intravenous immunoglobulin. MEP, methylprednisolone. MMF, mycophenolate mofetil.

### 3.2 Case report

#### 3.2.1 Case 1

A 65-year-old male patient was admitted to our neurologic intensive care unit (NICU) due to stiffness and spasms in both lower limbs lasting two weeks and a decrease in blood oxygen saturation for four days. Two weeks prior, without any obvious triggers, he developed weakness, tremors, stiffness, and swelling in both lower limbs, with symptoms progressing from distal to proximal regions. One week prior, abdominal stiffness appeared, accompanied by low back pain. He experienced spasms triggered by touch or mild auditory stimuli for several seconds. Treatment with clonazepam, baclofen, and levetiracetam at a local hospital failed to alleviate symptoms, which progressively worsened. Four days prior to admission, he suddenly developed generalized muscle spasms and a drop in blood oxygen saturation. The patient was immediately intubated. The patient had a history of a frontal head injury more than 10 years ago, with no sequelae. He denied any history of dog bites and had not received any vaccinations in the past six months. His other medical history was unremarkable.

On physical examination, the patient exhibited generalized rigidity and increased muscle tone in all limbs. Myoclonus was induced by mild stimulation, and hyperactive tendon reflexes were observed. The Babinski sign was positive on the left side. Routine laboratory tests revealed elevated white blood cell count (13.74*109/L; normal: 3.5-9.5*109/L), erythrocyte sedimentation rate (116 mm/H; normal: 0–20 mm/H), and C-reactive protein levels (40.85 mg/L; normal 0–6 mg/L). CSF analysis showed a slightly increased white cell count (13×10^6/L). CSF protein concentration was normal (352 mg/L). Immunological testing revealed positive serum anti-SS-A, anti-Ro-52, and anti-M2 antibodies, while serum ANA, ANCA, autoimmune encephalitis antibodies, and demyelination-related antibodies (aquaporin-4 [AQP4], Myelin Oligodendrocyte Glycoprotein [MOG], and glial fibrillary acidic protein [GFAP]) were negative. Both serum and CSF antineuronal antibodies (Hu, Yo, Ri, CV2, amphiphysin, Ma2/Ta) were negative, as were tumor markers. However, anti-GlyR antibody (titer of 1:10) were detected in both blood and CSF. Brain magnetic resonance imaging (MRI) showed bilateral frontal lobe encephalomalacia lesions. The EEG background rhythm consisted of diffuse slow waves, and the clinical manifestations of tonic-clonic seizures were not correlated with the EEG changes. We performed chest computed tomography (CT), abdominal CT, serum paraneoplastic antibodies, and serum tumor markers to screen for hidden tumors, all of which yielded negative results.

Treatment with a combination of intravenous immunoglobulin (IVIg) at 0.4 g/kg/day and methylprednisolone at 80 mg followed by tapering oral prednisone were started. At day 14 of hospitalization, mycophenolate mofetil (MMF) was started. The patient’s response to immunotherapy was good. His symptoms were almost disappeared on the 12th day after admission. Electromyography (EMG) on the 20th day after admission indicated a small amount of motor unit potential (MUP) in the gastrocnemius muscle of the lower limbs, with either continuous or intermittent discharge. Before discharge, repeat testing showed a serum GlyR antibody titer of 1:30.

#### 3.2.2 Case 2

A 68-year-old man presenting with a 12-day history of trismus and dysphagia. He had difficulties opening the mouth, swallowing, and speech. Additional symptoms comprised alalia, dribbling, and bucking. His symptoms progressively worsened. He had not eaten for two days before admission. He had a history of cerebral infarction more than 10 years ago but denied any history of dog bites. No other significant medical history was reported.

On neurological examination, the patient was conscious, with slurred speech and hoarseness. Stimulation elicited stridor. CSF analysis identified plasma cells with normal cell count, slightly increased protein (0.503 g/l, normal: 0.15-0.45g/L), increased glucose (7.21mmol/L, normal: 2.5-4.5mmol/L), and decreased chloride (117.2mmol/L, normal: 120-132mmol/L). CSF testing revealed positive GlyR antibodies (titer of 1:10), while serum GAD65 (titer 1:10) and GlyR antibodies were also positive (titer of 1:30). Additionally, serum MOG antibodies were positive (titer of 1:10). Other autoimmune encephalitis antibodies (including antibodies against N-methyl-D-aspartate receptor (NMDAR), α‐amino‐3‐hydroxy‐5‐methyl‐4‐isoxazolepropionic acid receptor 1 (AMPAR1), AMPAR2, leucine-rich glioma-inactivated protein 1 (LGI1), contactin associated protein 2 (CASPR2), γ‐amino butyric acid type B receptor (GABABR), IgLON5, dipeptidyl-peptidase-like protein (DPPX), D2 dopamine receptor (DRD2), mGluR5, mGluR1, Neurexin-3α, GABAAR, Kelch-like protein 11 (KLHL11), and AchR), demyelination-related antibodies (AQP4, MOG, and GFAP), and antineuronal antibodies were negative both in blood and CSF. The EEG showed diffuse slow waves. Abdominal ultrasound, chest CT, and brain MRI with and without contrast showed no significant abnormalities. Spinal MRI and EMG was not performed.

The patient subsequently received immunotherapies with IVIg at 0.4 g/kg/day and methylprednisolone at 500 mg with a tapered dose. On the 25th day of hospitalization, the patient experienced worsening respiratory distress and significant laryngospasm, leading to respiratory failure and requiring endotracheal intubation. The patient’s family declined active therapy and he was discharged immediately. The patient finally passed away at home after their family removed the endotracheal tube.

#### 3.2.3 Case 3

A 58-year-old male patient presented to NICU with a 6-day history of rigidity and spasms in both lower limbs. Generalized myoclonic jerks elicited by sudden noise or touch, rendering him unable to walk. No dysphagia and dysarthria was reported. He had been previously diagnosed with acute myelitis at a local hospital but did not receive immunotherapy, and his symptoms progressively worsened with frequent episodes. His medical history included hypertension, diabetes, and a history of smoking. He denied any recent vaccination or history of dog bites.

On examination, the patient was conscious and fluent in speech, with a distressed expression. Muscle tone was elevated in both lower limbs, and hyperactive reflexes were observed. Tactile stimulation induced rigidity and clonus in both lower limbs and the trunk. Routine laboratory tests: blood routine, liver and kidney function tests, inflammatory markers, and thyroid function tests were all within normal limits. Extensive tumor screening, including chest CT, abdominal ultrasound, serum paraneoplastic antibody testing, and serum tumor marker assessment, was performed, but no evidence of malignancy was found. CSF routine examination revealed slightly elevated glucose levels (6.44mmol/L, normal: 2.5-4.5mmol/L), with all other parameters within normal limits. Antineuronal antibodies in serum and CSF were negative. MRI of the brain, cervical spine, and thoracic spine revealed no significant abnormalities. The EEG demonstrated diffuse slow waves. EMG of the limbs indicated peripheral nerve involvement, with axonal degeneration in some sensory nerve fibers and demyelination of motor nerve fibers and nerve roots, along with secondary axonal degeneration. Neurogenic abnormalities and early denervation changes were observed in the muscles of both upper and lower limbs, with a small amount of MUPs showing continuous or intermittent discharges. Both serum and CSF tested positive for anti-GlyR antibodies (titer of 1:10).

The patient showed significant improvement after receiving pulse therapy with methylprednisolone at 500 mg followed by a tapered dose, along with mycophenolate mofetil at 1,000 mg. The patient’s respiratory function remained unaffected throughout.

#### 3.2.4 Case 4

A 44-year-old woman presented with a 11-month history of startle-induced episodes characterized by generalized rigidity and painful spasms involving the face, trunk, and limbs, with shoulder pain over 20 days. Her symptoms had an insidious onset and progressed gradually. Initially, approximately 11 months prior, she experienced numbness and pain in the right side of her face. This was followed by sudden episodes of myoclonus and jerking movements, particularly affecting the axial muscles, which were highly sensitive to auditory stimuli and light touch. Each episode lasted only a few seconds, occurred spontaneously, and recurred up to dozens of times per day. This was her first hospitalization, during which local doctors provided symptomatic treatment, but her symptoms did not improve. After discharge, her condition progressively worsened. The frequency of these attacks rendered her unable to walk independently. She also reported poor sleep quality, persistent diplopia that led her to prefer keeping her eyes closed, a soft voice, and difficulty performing routine activities such as rolling over in bed or combing her hair. She was admitted to an inpatient clinic again. At that time, her symptoms had resolved almost completely following a course of temporary corticosteroid treatment.

Eight months earlier, she had been hospitalized for the third time for similar symptoms, including numbness on the right side of her face, myoclonic jerks in the right face and limbs triggered by light touch, and significant horizontal gaze impairment. After receiving methylprednisolone therapy and sequential tapering, her symptoms significantly improved. However, more than half a month after discontinuing oral prednisone, the previous symptoms reappeared, accompanied by marked shoulder pain, leading to the fourth hospitalization. Her past medical history was notable for MG associated with thymoma, diagnosed 8 years ago. The thymoma had invaded the capsule; therefore, thymectomy was performed, followed by 35 sessions of radiotherapy. Since then, she had been on a maintenance dose of pyridostigmine bromide 60 mg/day.

On physical examination, the patient appeared to be in pain, with an abnormal posture and a crooked back due to back pain. She exhibited a soft voice but no dysarthria. Notably, gaze-evoked nystagmus was observed on lateral gaze, particularly on rightward gaze. Prominent paroxysmal limb rigidity was noted, along with a positive Babinski response. Tendon reflexes were normal in all limbs, and finger-to-nose and heel-to-shin tests were negative. The remainder of the neurological examination was unremarkable.

Routine laboratory tests yielded unremarkable results, including assessments of thyroid function, serum ammonia, lactic acid, creatine kinase, ceruloplasmin, and urinary copper. Immunological evaluation showed an increased percentage of CD8-positive T lymphocytes and a decrease in CD4-positive T lymphocytes. Repeated CSF analysis revealed the presence of plasma cells with a normal cell count and no evidence of infectious agents, but intrathecal IgG synthesis was observed. Tests for acetylcholine receptor (AchR) and Titin antibodies were strongly positive (>20.0 nmol/L and 18.6 U/mL, respectively). Screening for antineuronal antibodies in blood and CSF was negative. Additionally, autoantibodies associated with central nervous system demyelination (AQP4, MOG, MBP), encephalomyelitis/encephalitis (IgLON5, DPPX, DRD2, mGluR5, mGluR1, neurexin-3α, NMDA, AMPA1, AMPA2, LGI1, GABAb, CASPR2), and gangliosides (GM1, GM2, GM3, GM4, GD1a, GD1b, GD2, GD3, GQ1b, GT1a, GT1b, sulfatide) were undetectable in both blood and CSF, even on two separate occasions. Comprehensive tumor screening found no abnormalities. Imaging studies, including the brain, spine, shoulder, thymus, chest, and abdomen, as well as EMG, were all normal. However, both anti-GAD65 antibodies (titer 1:30) and anti-GlyR antibodies (titer 1:10) were detected in blood and CSF. No additional autoantibodies related to the SPS spectrum were identified.

The patient was treated with intravenous methylprednisolone (1,000 mg/day) for 5 days, followed by a tapering course of oral prednisone and clonazepam (7.5 mg/day). In addition, at the 2-month follow-up, the patient showed significant improvement, with undetectable serum GlyR antibodies. However, serum GAD65 antibodies remained detectable (titer 1:30). Over a 4-year follow-up period, the patient experienced no relapses and achieved a good recovery. Repeated testing showed serum GAD65 antibody titers fluctuating (1:10 to 1:30) during the first 2.5 years but eventually turned negative by the end of the 4-year follow-up.

## 4 Discussion

PERM shares several clinical features with SPS, such as rigidity, stimulus-sensitive spasms, myoclonus, hyperekplexia, and autonomic disturbances. However, it can be distinguished from classic SPS by the presence of additional brainstem and other neurological involvement (2, 6). The clinical classification is primarily based on the frameworks established by Meinck and Thompson (2002) (6) and Espay and Chen (2006), which define PERM as involving brainstem pathology alongside the axial or limb rigidity typical of SPS in its various forms. In 2008, Hutchinson et al. reported a case of PERM in a patient without GAD antibodies who demonstrated significant recovery following treatment with steroids, plasma exchange, intravenous immunoglobulin, and cyclophosphamide (7). Retrospective analysis of the patient’s sera, collected at disease onset and peak severity, revealed antibodies targeting GlyRα1 subunits expressed on the surface of transfected human embryonic kidney cells and capable of immunoprecipitating GlyR. Subsequently, additional cases of PERM associated with GlyR antibodies have been reported, characterized by varying combinations of stiffness, rigidity, excessive stimulus-evoked startle, brainstem involvement, and autonomic signs (11, 12).

The disease affects females more than males (10), and can occur at any age (13). However, in our case series, three of the four patients diagnosed with PERM were male. The commonest features are spasms/stiffness/rigidity/myoclonus, often painful, of the neck, trunk or limb muscles, which are associated with walking difficulties and frequent falls. Other symptoms of PERM are excessive startle, oculomotor disturbance, trigeminal, facial and bulbar disturbance, autonomic disturbance, cognitive impairment, and respiratory failure (4). The clinical features in our case series were in many respect to those previously reported. In addition to limb/trunk spasms and excessive startle responses, Case 2 exhibited bulbar disturbances (dysphagia, dysarthria, and trismus), while Case 4 showed trigeminal and facial disturbances (facial numbness) and oculomotor disturbances (diplopia). Cases 1 and 2 required endotracheal intubation due to respiratory failure. Cases 1, 2, and 3 were admitted to the ICU.

GlyR is considered a specific antibody. GlyRs belong to the superfamily of ligand gated ion channels and are pentameric proteins composed of two α and three β subunits. Glycine activation of the GlyR leads to an influx of Cl^-^ into the neurons and results in hyperpolarization of the membrane potential and reduced excitation. Patients with GAD antibodies were more likely to develop SPS or overlapping syndromes than patients with GlyR antibodies, who more often developed PERM or other symptoms (SPS-plus) (14). Meinck et al. investigated the presence of GAD antibodies in 13 patients with SPS, 9 patients with PERM, 279 patients with other neurological disorders, and 100 healthy controls (6). They reported that GAD65 antibodies were detected in approximately 80% of patients with SPS and PERM, compared with 5% of those with other neurological diseases and 1% of controls. But GlyR antibodies appear more pathological than GAD65 antibodies. Detection of GlyR antibodies may prove helpful in the diagnosis of patients with symptoms and signs that include ocular motor and other brainstem dysfunction, hyperekplexia, stiffness, rigidity, myoclonus and spasms. Most patients with PERM test positive for either GlyR or GAD antibodies, while a subset of patients exhibit the coexistence of both (4, 12, 15, 16). In Case 2 and Case 4, both anti-GAD65 antibody and anti-GlyR antibody were detected in blood.

GlyR antibodies are against cell surface antigens while GAD65 antibodies are against intracellular antigens. The coexistence of GlyR and GAD antibodies suggests that neuronal surface antibodies, perhaps to different targets, could determine the clinical phenotypes of patients with GAD antibody associated diseases. Although patients with GlyR antibodies had more severe neurologic deficits than patients with GAD65 antibodies, the outcome of patients with GlyR was better than that of patients with GAD65 antibodies (14). Possible reasons include: 1. Direct inhibition or internalization of the GlyRs but not a destructive process is likely the causes of the dysfunction. 2. Moreover, patients with GlyR antibodies were treated more aggressively (e.g. first-line immunotherapy combined with second-line or long-term oral immunotherapy) than those with other antibodies. 3. On the other hand, GlyR antibodies are against cell surface antigens while GAD65 antibodies are against intracellular antigens. Disorders associated with antibodies to cell surface antigens are usually more responsive to immunotherapy than those associated with intracellular antigens. That indicates presence and type of antibodies but not the clinical manifestations are an independent predictor of outcome. Nevertheless, two patients (Case 2 and 4) with mixed antibodies initially presented with mild clinical symptoms; ultimately, Case 2 developed respiratory failure, while Case 4 achieved complete remission. Therefore, the relationship between types of these antibodies and clinical status and prognosis, is complex. In fact, antibody titers were not reassessed in many reported cases following treatment. It is hypothesized that the titers of GAD65 and GlyR antibodies detected by CBA may have clinical significance. Nevertheless, the exact correlation between antibody titers and clinical presentation, prognosis, and relapse warrants further confirmation in large-scale cohort studies. Longitudinal monitoring of antibodies levels in treated patients may help to clarify their clinical significance. Moreover, antibody testing in China started relatively late, leading to delayed recognition of this disease. In 2020, it took 11 months to diagnose in Case 4. However, in three cases diagnosed in 2022 (Cases 1, 2, and 3), the average time from symptom onset to diagnosis improved to 11.0 ± 1.63 days.

Comorbid autoimmune diseases are not uncommon (4, 17). Commonly associated autoimmune diseases include autoimmune thyroiditis, insulin-dependent diabetes mellitus, vitiligo, Sjögren’s syndrome, lupus, celiac disease, MG, rheumatoid arthritis, sarcoidosis, and mixed connective tissue disease (4, 17). Interestingly, in Case 4, PERM happened after thymectomy in MG patient. Both PERM and MG belong to autoimmune diseases, which indicates autoimmunity plays a major role in the pathogenesis of these neuromuscular disorders. MG is often associated with other autoimmune disorders such as autoimmune thyroid disease, autoimmune encephalitis, inflammatory muscle disease, Sjögren’s syndrome, systemic lupus erythematosus, etc (18, 19). In recent years, cases of MG combined with SPS or PERM have been reported (20–24). The coexistence of GAD65, GlyR, AchR, and Titin antibodies in this patient suggests the presence of multiple immune responses. In thymoma patients, defective self-immune T cells outputted from the thymus may persist long-term in the central and peripheral nervous systems, potentially triggering these autoimmune diseases (25, 26). Thymectomy as a treatment for MG may be a triggering factor for PERM, as thymectomy can potentially disrupt the balance of T cell regulatory activity. The treatment of MG and PERM also shares some similarities, requiring the use of steroids and immunosuppressants. However, the relationship between thymoma, MG, and PERM still requires further research for confirmation. In addition, clinical physicians should bear in mind that the clinical features of autoimmune diseases can overlap. If patients experience unexpected changes in their condition, demonstrate a poor response to the initial treatment regimen, or develop new symptoms or signs, it is crucial to consider the possibility of concurrent autoimmune diseases.

Retrospective studies indicate that approximately 20% of patients have concurrent tumors, such as lung cancer, thymoma, lymphoma, melanoma, metastatic tumors, and leukemia (4). For instance, Case 4 was complicated by thymoma, highlighting the need for regular tumor screening. Treatments include symptomatic, first-line immunotherapies, second-line immunotherapies, and long-term oral immunotherapy. Diazepam and clonazepam are used for relief of painful spasms. Immunotherapies, including steroid, IVIg, plasma exchange (PE) and rituximab, are the core of the treatment. Involvement of autonomic and respiratory systems is the main cause of unexplained deaths (4). Respiratory failure have contributed to the death of Case 2.

There were some limitations in the study. First, antibodies were not re-evaluated in Case 2 and Case 3. However, based on the findings from Case 1 and Case 4, it appears that GlyR antibody titers may correlate positively with clinical symptoms, which requires further validation with larger sample sizes. Second, early EMG was not performed. Due to ICU constraints, Case 2 did not undergo EMG, and the examination in Case 1 was only completed 20 days after admission, more than one month after symptom onset. Third, the number of cases in this series is relatively small. Future cohort studies are needed to comprehensively analyze the clinical characteristics of this condition. Last, this study was conducted as a retrospective observational analysis, and therefore the findings should be interpreted as associations rather than definitive causal relationships.

## 5 Conclusion

We describe a case series of four cases of PERM from China, summarizing the clinical and immunological characteristics of PERM from our center. Detection of GlyR antibodies may prove helpful in the diagnosis of patients with symptoms and signs that include stiffness, rigidity, myoclonus, spasms and ocular motor and other brainstem dysfunction. Given that most of the patients have a good clinical response to immunosuppressive therapies, timely diagnosis and intervention for PERM are paramount.

## Data Availability

The datasets presented in this article are not readily available because of ethical and privacy restrictions. Requests to access the datasets should be directed to the corresponding author/s.
